# Tumor-targeting cell-penetrating peptide, p28, for glioblastoma imaging and therapy

**DOI:** 10.3389/fonc.2022.940001

**Published:** 2022-07-22

**Authors:** Sunam Mander, Samer A. Naffouje, Jin Gao, Weiguo Li, Konstantin Christov, Albert Green, Ernesto R. Bongarzone, Tapas K. Das Gupta, Tohru Yamada

**Affiliations:** ^1^ Department of Surgery, Division of Surgical Oncology, University of Illinois College of Medicine, Chicago, IL, United States; ^2^ Department of Electrical and Computer Engineering, University of Illinois College of Engineering, Chicago, IL, United States; ^3^ Richard & Loan Hill Department of Biomedical Engineering, University of Illinois College of Engineering, Chicago, IL, United States; ^4^ Department of Anatomy and Cell Biology, University of Illinois College of Medicine, Chicago, IL, United States

**Keywords:** NIR fluorescence image, image-guided surgery, cell-penetrating peptide, glioblastoma, targeted therapy

## Abstract

Despite recent advances in cancer research, glioblastoma multiforme (GBM) remains a highly aggressive brain tumor as its treatment options are limited. The current standard treatment includes surgery followed by radiotherapy and adjuvant chemotherapy. However, surgery without image guidance is often challenging to achieve maximal safe resection as it is difficult to precisely discern the lesion to be removed from surrounding brain tissue. In addition, the efficacy of adjuvant chemotherapy is limited by poor penetration of therapeutics through the blood-brain barrier (BBB) into brain tissues, and the lack of tumor targeting. In this regard, we utilized a tumor-targeting cell-penetration peptide, p28, as a therapeutic agent to improve the efficacy of a current chemotherapeutic agent for GBM, and as a carrier for a fluorescence imaging agent for a clear identification of GBM. Here, we show that a near-infrared (NIR) imaging agent, ICG-p28 (a chemical conjugate of an FDA-approved NIR dye, indocyanine green ICG, and tumor-targeting p28 peptide) can preferentially localize tumors in multiple GBM animal models. Moreover, xenograft studies show that p28, as a therapeutic agent, can enhance the cytotoxic activity of temozolomide (TMZ), one of the few effective drugs for brain tumors. Collectively, our findings highlight the important role of the tumor-targeting peptide, which has great potential for intraoperative image-guided surgery and the development of new therapeutic strategies for GBM.

## Introduction

Glioblastoma multiforme (GBM) is the most frequent primary central nervous tumor with the characteristics of highly aggressive growth, high recurrence rate, and poor prognosis in adults (1). Current standard therapies include maximal safe resection, followed by radiotherapy plus concomitant treatment and maintenance with temozolomide (TMZ). Although there have been recent advances in treatment strategy, the prognosis of GBM still remains poor with a median survival of 14–16 months (1–3). There are several reasons why it is difficult to treat GBM: i) in general, GBM has various dysregulated molecular pathways due to numerous gene alterations that are difficult to block simultaneously with a monotherapy (4), ii) GBM is a highly heterogeneous tumor that displays a stem cell phenotype with characteristics of drug resistance (5–8), and iii) the blood-brain barrier (BBB) blocks most molecules (therapeutics agents) from entering the brain through blood vessels. Also, the surgical approach without any image guidance presents challenges as it is difficult to discern the true tumor margins to be resected from surrounding brain tissue to minimize the loss of brain function. Based on these characteristics, there are clinical needs to develop new approaches for the treatment of GBM.

We have previously demonstrated that a redox protein azurin secreted by an opportunistic pathogen *Pseudomonas aeruginosa* and its derived cell-penetrating peptide p28 preferentially enter various cancer cells and induce antiproliferative effects (9–13). Clinically, p28 (NSC745104) as a single therapeutic agent was tested in two phase I clinical trials and granted the FDA Orphan Drug and Rare Pediatric Disease designations as it demonstrated preliminary efficacy without apparent adverse effects, toxicity, or immunogenicity in pediatric patients with recurrent and refractory central nervous system tumors (NCI and Pediatric Brain Tumor Consortium) and in adult patients with advanced solid tumors (14–16). Recently, we reported that a new NIR optical imaging probe, ICG-p28, composed of clinically nontoxic p28 and FDA-approved ICG can be used as an intraoperative imaging agent in breast tumors animal models (17). Image-guided surgery with ICG-p28 clearly defines margins in a real-time, and 3D fashion, resulting in adequate tumor excision in preclinical settings (17, 18). As such, p28 is a potential therapeutic agent as well as a tumor-targeted carrier molecule since it preferentially penetrates cancer cells, is highly water-soluble and stable, and clinically exhibits no significant adverse effects, toxicity, or immunogenicity in humans. Here, we show characteristics of p28 penetration in GBM and the therapeutic potential of p28 in combination with a standard chemotherapeutic agent for GBM.

## Materials and methods

### Peptide synthesis and conjugation

p28 peptide was synthesized by CS Bio, Inc. at >97% purity and mass balance. Peptide labeling with Alexa fluor 568 and ICG was described before (11, 17). Gold nanoparticles (GNPs) (Nanopartz Inc., CO; #CS11-15) were conjugated with p28 according to the manufacturer’s instructions. Briefly, NHS (N-hydroxysuccinimide) esters-functionalized spherical GNPs (15 nm) were conjugated with a five hundred molar excess of p28 in 0.1 M borate buffer (pH 8.0) at room temp for 4 hr. The labeled peptide was washed with 1% PBS (Phosphate-buffered saline)/0.1% Tween 20 at 9,000 rcf for 10 min and resuspended in PBS. Gold nanoparticles alone (Nanopartz Inc; #C11-15-NC-5) were used as a control.

### Cell cultures

The human glioblastoma cell lines (LN-229 and U-118) and astrocytes and fibroblasts were obtained from American Type Culture Collection (Manassas, VA). Astrocytes were cultured in Astrocyte Growth Media Bullet Kit (Lonza, Walkersville, MD). Others cell lines were cultured in MEME (Minimum Essential Medium Eagle) supplemented with 10% fetal bovine serum at 37°C in a humidified incubator with 5% CO_2_.

### Confocal microscopy

Cells were seeded overnight on glass coverslips at 37°C under 5% CO_2_, rinsed with fresh medium, and incubated at 37°C for 2 h in prewarmed medium containing Alexa Fluor 568–labeled p28 at 20 μM (9, 11, 19, 20). After washing, coverslips were mounted in medium containing 1.5 μg/mL 4′,6-diamidino-2-phenylindole (DAPI) to counterstain nuclei (VECTASHIELD; Vector Laboratories). Cellular uptake and distribution were imaged by a confocal microscope (Meta 710, Carl Zeiss Inc.).

### Native gel electrophoresis

LN-229, U-118, and fibroblast were exposed to 20 μM of ICG-p28 for 2 hr. After washing with PBS twice, cell lysates were made by glass homogenizer followed by centrifugation at 13,000 rpm for 15 min at 4°C. Lysates were loaded (20 µg/lane) on 10% Native PAGE gels. ICG-p28 in gels was scanned by the Odyssey imaging system (Li-cor) with the 800 nm channel. Positive controls at 10, 20, and 50 ng/lane of pure ICG-p28 were used.

### Transmission electron microscopy

LN-229 cells were exposed to p28-GNRs (300 µg/ml) for 2 hr. Cell culture samples (~1 mm^3^) were fixed in 4% phosphate-buffered glutaraldehyde, followed by washing samples with 2% sucrose in 0.1 M Sorensen’s phosphate buffer at room temp. Samples were treated with 1% osmium tetroxide in 0.1 M Sorensen’s phosphate buffer for 1 hr. After washing with dH_2_O and dehydration with acetone, resin infiltration (EMBed 812 resin [EMS]) was performed; 2:1 mix of 100% acetone:resin, 1:1 mix of 100% acetone:resin, 1:2 mix of 100% acetone:resin, and 100% resin. Samples were placed in embedding molds and polymerized. Sections (60 nm) were made using an Ultratome and picked up on 200 mesh copper grids. TEM images were taken with a JEOL 1220.

### Cytotoxicity assay

Cytotoxicity was evaluated by the MTT assay as described previously (21, 22). Cells seeded in 96-well plates were exposed to TMZ at various concentrations for 72 hr (N=3 in each concentration). Curves were fitted using GraphPad PRISM (version 5.0) as a function of a nonlinear one-phase decay (maximum iterations: 1,000, Cl: 95%).

### Animal models

All animal work was performed under a protocol approved by the University of Illinois at Chicago. Athymic mice at 4-5 weeks old were purchased from The Jackson Laboratory. For orthotopic xenografts, GBM cell lines (LN-229 and U-118) were injected into the cerebral cortex. Following anesthesia, cancer cell suspensions at 5x10^5^ cells/3μl were stereotactically injected (2 mm lateral to the sagittal suture, 1 mm anterior to the coronal suture, 3 mm depth) using a Koft model 963 stereotactic apparatus with a 31-gauge Hamilton syringe. After injection, the injection site was covered with sterile bone wax. Tumor growth was monitored by MR imaging. For tumor growth study, GBM cell lines (LN-229 and U-118) at 1.0x10^6^ cell/mouse were subcutaneously (*s.c.*) implanted into the right flank of athymic mice (22). When tumors reached 3 mm in diameter, animals were randomized into several groups; PBS (control), TMZ 5 mg/kg (25.7 μmol/kg) 5 injections on Day1-5, and p28 10 mg/kg (3.4 μmol/kg) 3 times weekly, *i.p*. Body weight was determined 3 times weekly. The tumor volume in each animal was assessed by calipers and calculated 3 times weekly.

### MR imaging

MR images of the mouse brain with or without a contrast agent were recorded by a 9.4T MRI system (Agilent, Santa Clara, CA). Mice were anesthetized with isoflurane, temperature was maintained and respiration was monitored throughout the entire scan. T_2_-weighted MR images were acquired using a fast spin-echo sequence with the following acquisition parameters: TR/TE 2050/8 ms, echo train length 8, matrix 128 × 128, FOV 19.2 mm × 19.2 mm, slice thickness 1 mm. T_1_-weighted MR images were acquired using a spin-echo sequence with the following acquisition parameters: TR/TE 500/11.52 ms, matrix 192 × 192, FOV 19.2 mm × 19.2 mm, slice thickness 1 mm. Prior to the T_1_-weighted MRI acquisition, contrast administration (Magnevist, gadopentetate dimeglumine at 0.2 mM/kg) was injected in the tail vein.

### NIR fluorescence imaging

Mice bearing tumors in the brain (N=3 in each cell line) were injected with ICG labeled p28 at 0.5 mg/kg. After 24 hr, the brains were scanned by the Odyssey imaging system (17). Specific NIR signals at 700 and 800 nm were recorded. The tumor background ratio was determined using ImageJ software (National Institutes of Health), where a region of interest (ROI) was drawn over the tumor and surrounding brain tissue. An ROI was manually generated around each tumor (800 nm channel).

### Histological analysis

Resected tumors were fixed with buffered 3.7% formalin (Anatech) for 24 hr. Formalin was replaced with 70% ethanol following fixation. Samples were paraffin-embedded, and blocks were cut into 4 μm-thick sections and mounted onto slides. H&E- and TUNEL-stained slides were analyzed by a pathologist blinded to the experimental groups and tumor growth results.

### Statistical analysis

Data processing and statistical analysis were performed using GraphPad Prism. ANOVA test was performed for tumor growth analysis, and P<0.05 was considered statistically significant.

## Results

### Preferential cellular entry of p28

**Figure 1:**
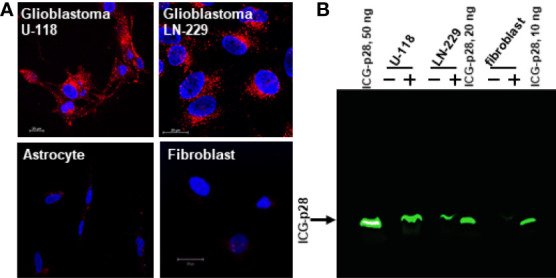
Preferential penetration of p28 into human glioblastoma cells. **(A)**, human GBM cell lines (U-118 and LN-229) and astrocyte and fibroblast were cultured with Alexa Fluor 568–labeled p28 at 37°C for 2 hr, and images were recorded by confocal microscopy. Red, Alexa Fluor 568–labelled p28; blue, DAPI (nucleus). **(B)**, Human GBM (LN-229 and U-118) and fibroblast were treated with ICG-p28 for 2 hr. Lysates (20 µg/lane) were separated by 10% Native PAGE and ICG-p28 was imaged by the Odyssey imaging system with an 800nm channel. Positive control: 10, 20, and 50 ng/lane of pure ICG-p28. +: ICG-p28 treated cell lysate. -: untreated cell lysate.

**Figure 2:**
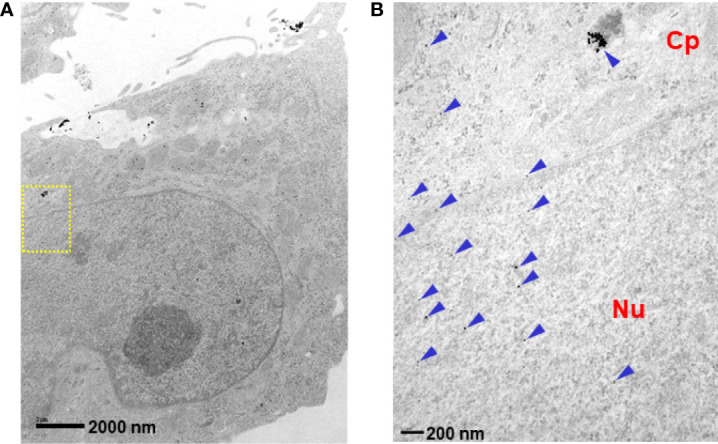
Intracellular localization of p28. LN-229 cells were treated with p28-GNRs for 2 hr. Sections (60 nm) of fixed cells on 200 mesh copper grids were imaged. TEM images taken by a JEOL 1220 showed a significant amount of p28-GNP uptake (arrowheads). **(A)**: x10,000 magnifications, **(B)**: location of yellow squire at x40,000. Cp: cytoplasmic space. Nu: nucleus.

We analyzed the cellular internalization properties of p28 in multiple human glioblastomas (GBM) cell lines. Confocal images of cells exposed to Alexa Fluor 568 labeled p28 showed that p28 preferentially entered GBM cell lines, U-118 and LN-229 whereas minimal signal was detected from astrocytes and fibroblasts treated with the same concentration of the probe (**Figure 1A**). To confirm, GBM and fibroblasts were exposed to ICG fluorescent dye (indocyanine green) labeled p28. Cell lysates of GBM also showed p28 preferential penetration and retained intact p28 molecule in GBM (**Figure 1B**). These two independent experiments demonstrated preferential internalization. As we used two different probes (Alexa Fluor 568 and ICG), the effect was due to p28, not fluorescent dyes. Furthermore, transmission electron microscope (TEM) images were recorded to finely visualize the cellular localization of p28. Gold nanoparticles have been used as intracellular imaging agents, and their cellular uptake as well as nuclear entry of gold nanoparticles are size and shape-dependent (23, 24). Small nanoparticle itself (*e.g*., <10 nm) can efficiently penetrate tumors (23) and enter the nucleus (24). Since our objective in this study was to determine the effect of p28 as a targeted-delivery vehicle, we used relatively large size of gold nanoparticles with a diameter of 15 nm. Human GBM LN-229 cells were exposed to gold-nano particle (GNP) alone or GNP-labeled p28. TEM images showed very little entry of 15 nm GNPs alone (**Supplementary Figure 1**). In contrast, LN-229 cells exposed to GNP-labeled p28 showed substantial cellular penetration of GNP-labeled p28 (**Figure 2A**). A higher magnification TEM image showed that p28 was distributed in cytoplasmic space and nuclear. Also, endosomal localization of p28 was found in LN-229 cells (**Figures 2A, B** **)**.

### Preferential tumor localization of p28

**Figure 3:**
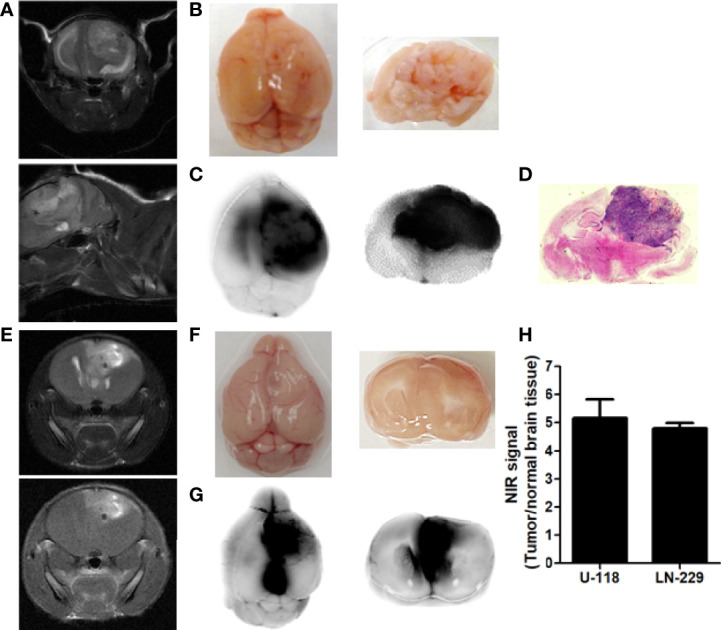
Preferential localization of p28 at tumor in the brain. MR images of the brains indicated U118 **(A)**, coronal (upper) and sagittal (lower) sections of T_2_-weighted images) and LN-229 tumor growth (**E**: upper: coronal section of T_2_-weighted image. lower: T_1_-weighted image) in the brain. Mice bearing tumor received ICG, a near-infrared red fluorescent dye conjugated with p28 at 0.5 mg/kg *i.v.* After 24h, fluorescence images of the brain were recorded **(B, F)**: white light photo, **(C, G)**: NIR fluorescence). Specific NIR signal at 800 nm was represented as direct fluorescence showing preferential localization of p28 at tumors. **(D).** The paraffin-embedded brain sections were stained by H, E. **(H),** The signal-to-background ratio (signal at 800 nm: tumor, background noise: surrounding brain tissue) was calculated. N=3 in each cell line, Mean+SD.

To determine whether p28 can localize at tumors in the brain, we assessed the *in vivo* localization of p28 in GBM orthotopic xenografts mouse models. U118 cells were implanted in the mouse brain. Tumor growth was confirmed by T_1_-weighted MRI with gadolinium-based contrast agent and T_2_-weighted MRI (**Figure 3A**). To visualize p28, ICG-p28 at 0.5mg/kg was injected systemically (tail vein, *i.v*.). NIR fluorescence images showed that p28 preferentially localized within U118 tumor correlates to MR images (**Figures 3B, C** **)**. *Ex vivo* images showed a good overlap between ICG-p28 fluorescence and H&E staining (**Figure 3D**). Similar to the animal model with U-118, LN-229 tumor development in the brain was confirmed by MIR (**Figure 3E**). NIR fluorescence images showed p28 preferential localization at LN-229 GBM (**Figures 3F, G** **)**. The NIR signal intensities from both U-118 and LN-229 tumors were substantially higher when compared to normal brain tissue (**Figure 3H**). These results illustrate the preferential uptake of p28, which is retained at the tumor site in the brain and makes clear contrast imaging of tumor vs. surrounding brain tissue.

### Effects of temozolomide in GBM cells

**Figure 4:**
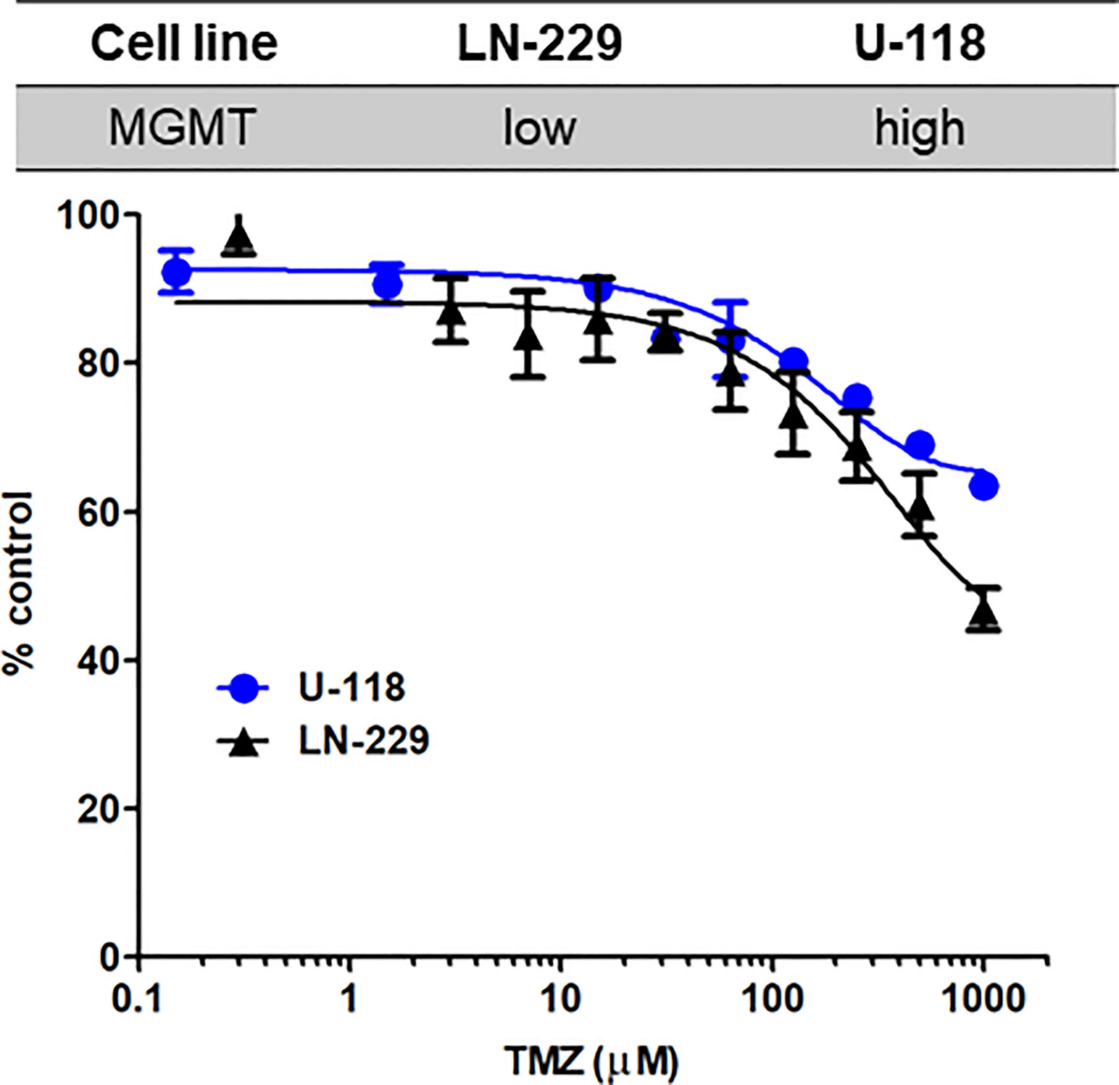
Susceptibility to TMZ in GBM cells with different MGMT expression levels. LN-229 (black triangle) and U-118 (blue circle) cells were exposed to various concentrations of TMZ for 72 hr. Dose-response curves for TMZ were determined by MTT assays. N=3 in each concentration. Mean ± SD.

Temozolomide (TMZ)-based chemotherapy is a part of the current standard treatment for adult GBM in the first-line and recurrent settings (25, 26). TMZ is an S_N_1 alkylating agent that induces DNA damage and cell death (27, 28). We first determined the effects of TMZ in LN-229 and U-118 (**Figure 4**). Relatively high doses of TMZ were needed to produce substantial antiproliferative effects. TMZ at 1 mM showed 53.2% and 36.6% reduction in LN-229 and U-118, respectively (P<0.001, two-tailed t-test). LN-229 cells were more susceptible to TMZ than U-118 cells. As TMZ is an alkylating DNA damaging agent, TMZ resistance can be mediated by levels of endogenous DNA repair enzyme. It has been reported that U-118 cells highly express O^6^-methylguanine-DNA methyltransferase (MGMT), a DNA repair enzyme, whereas LN-229 cells express low levels of MGMT (29). This is also consistent with clinical evidence that MGMT plays an important role in chemoresistance to alkylating agents (30, 31).

### Growth inhibitory effect of p28

**Figure 5:**
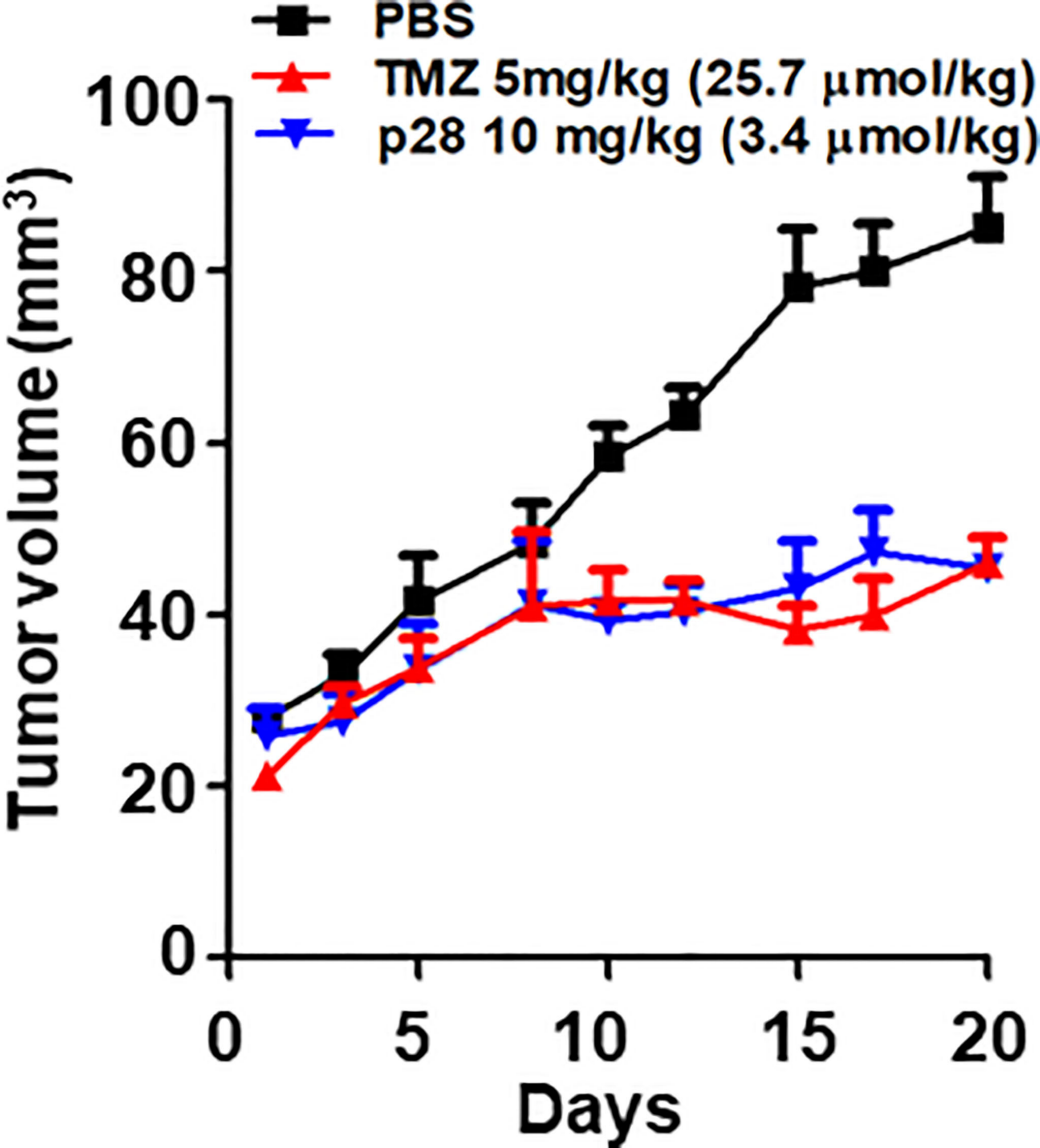
Antitumor effect of TMZ alone and p28 alone in a xenograft tumor model. Athymic mice bearing human GBM LN-229 cells were randomized into three groups; PBS (control), TMZ 5 mg/kg (=25.7 μmol/kg), and p28 10 mg/kg (=3.4 μmol/kg) *i.p.* N=5/group, Mean+SD.

We previously reported that p28 alone can induce significant antiproliferative effects on GBM cell lines *in vitro* (22). Here, we examined the anti-tumor effects of p28 and TMZ on LN-229 xenograft mice. p28 treatment significantly inhibited LN299 tumor growth *in vivo* (**Figure 5**). On day 20, TMZ treatment produced 42.4% reduction in tumor growth compared to the PBS control group (P<0.001, ANOVA). Tumor growth inhibition by p28 (46.5% vs. PBS, P<0.001, ANOVA) was similar to the effect of TMZ (P>0.05, p28 vs. TMZ). These results showed the significant inhibitory effect of p28 in the animal model.

### p28 improves the activity of a chemotherapeutic agent, TMZ

**Figure 6:**
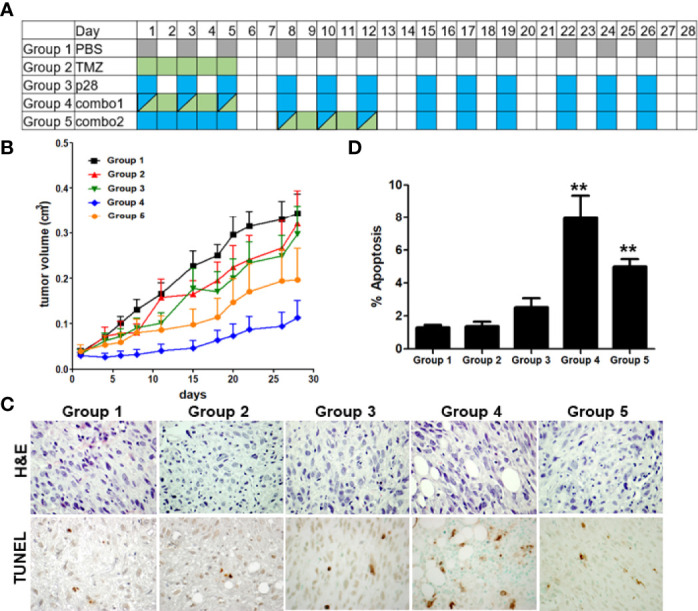
Improvement of the TMZ activity in combination with p28. **(A),** Treatment schedule of Groups 1-5. Athymic mice bearing human GBM U-118 cells were randomized into five groups; PBS (control), TMZ 5 mg/kg (=25.7 μmol/kg), p28 10 mg/kg (=3.4 μmol/kg) *i.p*., and combinations. **(B),** U-118 xenograft tumor growth curve. N=7/group, Mean+SD. **(C).** Formalin-fixed tumor sections were stained with H&E and TUNEL (apoptosis). **(D).** TUNEL-positive cells were counted in each group. **P<0.01 (ANOVA).

It has been suggested that p28 can improve the activity of DNA-damaging agents *in vitro* (22). To investigate whether the effects of p28 observed *in vitro* can translate into *in vivo* effects, we further examined whether p28 can enhance the activity of TMZ in U-118 cells that highly expressed MGMT and were less susceptible to TMZ (**Figure 4**). The inhibitory effect of p28 in combination with TMZ was directly compared to PBS control (Group 1), TMZ alone (Group 2), and p28 alone (Group 3) (**Figure 6A**). As compared to **Figure 5**, TMZ produced only a 24.5% reduction of U-118 tumor growth on day 20, confirming that U-118 was less susceptible to TMZ treatment (**Figure 6B**). On the other hand, the inhibitory effect of p28 alone was relatively similar in both LN-229 and U-118 xenografts (a 32.6% reduction in U-118 at day 20). Clinically, the approved conventional schedule for TMZ is a daily dose of 150-200 mg/m^2^ for 5 days of every 28-day cycle (32, 33). In combination groups, we had two different treatment schedules considering the current clinical treatment for TMZ; Group 4: co-administration of p28 and TMZ. Group 5: p28 treatment followed by TMZ. In group 5, tumor volume at day 28 was smaller than TMZ (38.6% reduction) and p28 (34.1% reduction) (Groups 1, 2, and 3 vs. Group 5, P>0.05, ANOVA). In contrast, the mice in group 4 (p28 and TMZ treatment at the same time) led to significantly greater inhibition of tumor growth (Groups 1, 2, and 3 vs. Group 4, P<0.05, ANOVA) without any loss in body weight (control group: 27.3 ± 0.3; TMZ: 29.0 ± 0.8; p28: 29.1 ± 0.7; combination: 29.5 ± 0.9 g) over the course of four weeks of *i.p*. treatment. The histological analyses of tumors indicated that only a few apoptotic cells were found in the tumor parenchyma of control animals (Group1) (**Figures 6C, D** **)**. Conversely, p28 in combination with TMZ (Groups 4, 5) showed a significant increase in apoptotic cells (**Figures 6C, D** **)**. There was a lack of cells in some tumor areas, suggesting tumor disintegration (**Figure 6C**). These data confirmed the *in vitro* data presented above and suggest that p28 inhibits tumor growth and it improves the TMZ activity when combined.

## Discussion

In this study, we demonstrated that p28 preferentially entered GBM and localized in cytoplasmic space and nucleus. *In vivo* imaging with ICG labeled p28 showed clear tumor localization and a significant contrast over the normal brain tissue. Upon entry, p28 induced a significant anti-tumor effect on GBM. Moreover, p28 enhanced the TMZ activity when they were combined in our GBM animal model.

The surgical treatment for GBM presents challenges due to the delicate tradeoff between removing tumors and minimizing the loss of brain function. Also, brain shift of as much as 1 cm can occur after craniotomy and dural opening (34) due to CSF egress, diminished mass effect, osmotic diuresis, edema, lesion resection, or intraoperative pneumocephalus (35, 36). These changes lead to preoperatively acquired images being inaccurate, limiting their usefulness in an operating room. To date, several intraoperative imaging techniques have been developed for brain tumor surgery (37). There are several intraoperative modalities available including MRI, ultrasound, and CT (38, 39). Intraoperative imaging allows visualization of brain shift and achieves a maximally safe tumor removal. While beneficial, each technique has advantages and disadvantages such as long scanning time and low image quality. Aside from these techniques, intraoperative optical imaging with 5-aminolevulinic acid (5-ALA) appears to be a new and promising technique in the field of malignant glioma treatment (37). Although image-guided surgery with 5-ALA represents one of the important advancements in the neurosurgical field, there are challenges to using this procedure. In normal cells, nonfluorescent 5-ALA is used for the porphyrin synthesis pathway that leads to heme synthesis. In malignant cells, 5-ALA as a prodrug is metabolized to fluorescent protoporphyrin IX (40). Since fluorescence intensity is depending on the metabolic activity in a tumor, image-guided surgery with 5-ALA is limited in regions with low-density tumor cell infiltration (41). Also, fluorescence in the tumor is typically hidden by photobleaching, blood, or overhanging brain tissue due to a relatively short wavelength of protoporphyrin IX excitation (λ_ex_ 380–440 nm, λ_em_ 620–640 nm) (42, 43). In contrast, NIR agents such as ICG are suitable because fluorescence intensity is independent of the metabolic activity in a tumor and their excitation is much longer (λ_ex_ 780 nm, λ_em_ 820 nm) (42), allowing deeper tissue penetration and a higher signal-to-noise ratio (“low-noise”) as intrinsic autofluorescence from tissues is quite low in this wavelength (44, 45). Thus, it is evident that a novel method using tumor-targeting NIR agent is critically needed in the operating rooms to help optimize the surgical excision that would significantly benefit patients.

The modes of cellular entry for p28 involve endocytosis and direct plasma membrane translocation (11, 20, 46). In general, direct membrane translocation is a single-step process in which CPPs cross the cellular lipid bilayer of cells (47–49). On the other hand, endocytosis is a well-established pathway of cellular entry for CPPs (50, 51). This mechanism of cellular entry involves endocytic uptake followed by endosomal escape (52). While positively charged (cationic) CPPs interact with the negatively charged heparan sulfate, anionic p28 entry involves caveolin-mediated endocytosis (11). Based on TEM results, p28 was distributed over two different but interconnected compartments, the cytoplasmic space and the nucleus. In the cytoplasmic space, p28 was entrapped in endosomes. Indeed, p28 in the lumen of endocytic organelles is topologically separate from the cytosolic space or other intracellular organelles such as the nucleus. It has been suggested that the addition of specific signal peptide sequences that interact with endosomal membranes or lead to the nucleus has been shown to alter the intracellular localization of CPPs (53, 54). Since endosomal entrapment is a major bottleneck, alternation of intracellular localization of p28 by inducing endosomal escape may substantially affect its degradation rate and/or activity as an imaging agent and therapeutic agent. The degradation of certain substrates requires movement into and out of the nucleus (55). For instance, p28 binds to the p53 DNA-binding domain and stabilizes it in cancer cells. In response to cellular stress, p53 as a transcription factor accumulates in the nucleus. Although nuclear proteins can be degraded by importing proteasomes into the nucleus, it is also evident that some nuclear proteins are degraded only after export to cytosolic proteasomes (55). Also, the lack of nuclear peptidase activity reportedly suggests that little degradation occurs in the nucleus (56). These observations suggest that alteration of the intracellular p28-distribution could substantially affect its activity. Further studies (*e.g.*, attaching specific signal peptides to p28) could define an important regulatory step in the p28 stability that should reflect the efficacy.

p28 can enhance the cytotoxic activity of DNA-damaging drugs such as TMZ. We demonstrated that the enhanced activity of TMZ in combination with p28 was facilitated through the p53/p21/CDK2 pathway (22). In our *in vivo* study (**Figure 6**), co-administration of p28 and TMZ (Group 4) showed more effectiveness than p28 treatment followed by TMZ (Group 5). It has been suggested that the combination therapy with anti-angiogenic drugs needs to be carefully designed in the schedule of drug administration (57). As p28 also has anti-angiogenic activity (58), it is possible that the p28 pre-treatment leads to a decrease in TMZ uptake. Based on our results, it will be appropriate to co-administer p28 and TMZ to maximal effects.

In summary, we demonstrated that p28 preferentially entered GBM tumor and showed a clear contrast over the normal brain tissue. Also, in a GBM animal model, p28 significantly enhanced the activity of the DNA-damaging agent TMZ when combined. Our findings support the future development of our promising approach through image-guided surgery with ICG-p28 and/or combination therapy with p28.

## Data Availability Statement

The original contributions presented in the study are included in the article/supplementary material. Further inquiries can be directed to the corresponding author.

## Ethics Statement

The animal study was reviewed and approved by University of Illinois at Chicago (UIC) Institutional Animal Care and Use Committee (IACUC).

## Author Contributions

Conceptualization, TG and TY. Study design, EB, TG, and TY. Data collection, SM, SN, JG, WL, KC, and AG. Data analysis, SM, KC, and TY. Writing draft and editing, SM, JG, TG, and TY. All authors reviewed, discussed, and agreed with the publication of the manuscript.

## Funding

This research was supported in part by the NIH/National Institute of Biomedical Imaging and Bioengineering (R01EB023924) and the NIH/National Cancer Institute (R21CA252370) to TY.

## Conflict of Interest

The authors declare that the research was conducted in the absence of any commercial or financial relationships that could be construed as a potential conflict of interest.

## Publisher’s Note

All claims expressed in this article are solely those of the authors and do not necessarily represent those of their affiliated organizations, or those of the publisher, the editors and the reviewers. Any product that may be evaluated in this article, or claim that may be made by its manufacturer, is not guaranteed or endorsed by the publisher.

## Supplementary Material

Supplementary Figure 1 TEM image of GBM cells exposed to GNRs alone. LN-229 cells were treated with GNRs alone for 2 hr. Sections (60 nm) of fixed cells on 200 mesh copper grids were imaged. TEM images taken by a JEOL 1220 showed very little GNP cellular entry

## References

[1] Ostrom QT Cioffi G Waite K Kruchko C Barnholtz-Sloan JS. Cbtrus statistical report: Primary brain and other central nervous system tumors diagnosed in the united states in 2014-2018. Neuro Oncol (2021) 23(12 Suppl 2):iii1–iii105. doi: 10.1093/neuonc/noab200 34608945 PMC8491279

[2] Stupp R Taillibert S Kanner A Read W Steinberg D Lhermitte B. Effect of tumor-treating fields plus maintenance temozolomide vs maintenance temozolomide alone on survival in patients with glioblastoma: A randomized clinical trial. JAMA (2017) 318(23):2306–16. doi: 10.1001/jama.2017.18718 PMC5820703 29260225

[3] Aldape K Brindle KM Chesler L Chopra R Gajjar A Gilbert MR. Challenges to curing primary brain tumours. Nat Rev Clin Oncol (2019) 16(8):509–20. doi: 10.1038/s41571-019-0177-5 PMC6650350 30733593

[4] Alifieris C Trafalis DT. Glioblastoma multiforme: pathogenesis and treatment. Pharmacol Ther (2015) 152:63–82. doi: 10.1016/j.pharmthera.2015.05.005 25944528

[5] Berger F Gay E Pelletier L Tropel P Wion D. Development of gliomas: potential role of asymmetrical cell division of neural stem cells. Lancet Oncol (2004) 5(8):511–4. doi: 10.1016/S1470-2045(04)01531-1 15288241

[6] Davis B Shen Y Poon CC Luchman HA Stechishin OD Pontifex CS. Comparative genomic and genetic analysis of glioblastoma-derived brain tumor-initiating cells and their parent tumors. Neuro Oncol (2016) 18(3):350–60. doi: 10.1093/neuonc/nov143 PMC4767234 26245525

[7] Singh SK Hawkins C Clarke ID Squire JA Bayani J Hide T. Identification of human brain tumour initiating cells. Nature (2004) 432(7015):396–401. doi: 10.1038/nature03128 15549107

[8] Nakada M Nakada S Demuth T Tran NL Hoelzinger DB Berens ME. Molecular targets of glioma invasion. Cell Mol Life Sci (2007) 64(4):458. doi: 10.1007/s00018-007-6342-5 17260089 PMC11138430

[9] Yamada T Goto M Punj V Zaborina O Chen ML Kimbara K. Bacterial redox protein azurin, tumor suppressor protein p53, and regression of cancer. Proc Natl Acad Sci U S A (2002) 99(22):14098–103. doi: 10.1073/pnas.222539699 PMC137843 12393814

[10] Yamada T Hiraoka Y Ikehata M Kimbara K Avner BS Das Gupta TK. Apoptosis or growth arrest: Modulation of tumor suppressor p53's specificity by bacterial redox protein azurin. Proc Natl Acad Sci U S A (2004) 101(14):4770–5. doi: 10.1073/pnas.0400899101 PMC387323 15044691

[11] Taylor BN Mehta RR Yamada T Lekmine F Christov K Chakrabarty AM. Noncationic peptides obtained from azurin preferentially enter cancer cells. Cancer Res (2009) 69(2):537–46. doi: 10.1158/0008-5472.CAN-08-2932 19147567

[12] Yamada T Das Gupta TK Beattie CW. P28, an anionic cell-penetrating peptide, increases the activity of wild type and mutated P53 without altering its conformation. Mol Pharm (2013) 10(9):3375–83. doi: 10.1021/mp400221r 23952735

[13] Yamada T Christov K Shilkaitis A Bratescu L Green A Santini S. p28, a first in class peptide inhibitor of cop1 binding to p53. Br J Cancer (2013) 108(12):2495–504. doi: 10.1038/bjc.2013.266 PMC3694247 23736031

[14] Warso MA Richards JM Mehta D Christov K Schaeffer C Rae Bressler L. A first-in-class, first-in-human, phase i trial of p28, a non-hdm2-mediated peptide inhibitor of p53 ubiquitination in patients with advanced solid tumours. Br J Cancer (2013) 108(5):1061–70. doi: 10.1038/bjc.2013.74 PMC3619084 23449360

[15] Razzak M. Targeted therapies: One step closer to drugging p53. Nat Rev Clin Oncol (2013) 10(5):246. doi: 10.1038/nrclinonc.2013.43 23507742

[16] Lulla R Goldman S Beattie CW Yamada T Pollack I Fisher P. Phase 1 trial of p28 (nsc745104), a non-hdm2 mediated peptide inhibitor of p53 ubiquitination in children with recurrent or progressive cns tumors: A final report from the pediatric brain tumor consortium. Neuro Oncol (2016) 18(9):1319–25. doi: 10.1093/neuonc/now047 PMC4999001 27022131

[17] Goto M Ryoo I Naffouje S Mander S Christov K Wang J. Image-guided surgery with a new tumour-targeting probe improves the identification of positive margins. ebiomedicine (2022) 76:103850. doi: 10.1016/j.ebiom.2022.103850 35108666 PMC8814381

[18] Naffouje SA Goto M Coward LU Gorman GS Christov K Wang J. Nontoxic tumor-targeting optical agents for intraoperative breast tumor imaging. J medicinal Chem (2022) 65(10):7371–9. doi: 10.1021/acs.jmedchem.2c00417 35544687

[19] Yamada T Goto M Punj V Zaborina O Kimbara K Das Gupta TK. The bacterial redox protein azurin induces apoptosis in j774 macrophages through complex formation and stabilization of the tumor suppressor protein p53. Infection Immun (2002) 70(12):7054–62. doi: 10.1128/iai.70.12.7054-7062.2002 PMC133031 12438386

[20] Yamada T Fialho AM Punj V Bratescu L Gupta TK Chakrabarty AM. Internalization of bacterial redox protein azurin in mammalian cells: entry domain and specificity. Cell Microbiol (2005) 7(10):1418–31. doi: 10.1111/j.1462-5822.2005.00567.x 16153242

[21] Goto M Yamada T Kimbara K Horner J Newcomb M Gupta TK. Induction of apoptosis in macrophages by pseudomonas aeruginosa azurin: Tumour-suppressor protein p53 and reactive oxygen species, but not redox activity, as critical elements in cytotoxicity. Mol Microbiol (2003) 47(2):549–59. doi: 10.1046/j.13652958.2003.03317.x 12519204

[22] Yamada T Das Gupta TK Beattie CW. p28-mediated activation of p53 in g2–m phase of the cell cycle enhances the efficacy of dna damaging and antimitotic chemotherapy. Cancer Res (2016) 76(8):2354–65. doi: 10.1158/0008-5472.CAN-15-2355 26921335

[23] Huang K Ma H Liu J Huo S Kumar A Wei T. Size-dependent localization and penetration of ultrasmall gold nanoparticles in cancer cells, multicellular spheroids, and tumors in vivo. ACS nano (2012) 6(5):4483–93. doi: 10.1021/nn301282m PMC3370420 22540892

[24] Huo S Jin S Ma X Xue X Yang K Kumar A. Ultrasmall gold nanoparticles as carriers for nucleus-based gene therapy due to size-dependent nuclear entry. ACS nano (2014) 8(6):5852–62. doi: 10.1021/nn5008572 PMC4076024 24824865

[25] Stupp R Hegi ME Mason WP van den Bent MJ Taphoorn MJ Janzer RC. Effects of radiotherapy with concomitant and adjuvant temozolomide versus radiotherapy alone on survival in glioblastoma in a randomised phase iii study: 5-year analysis of the eortc-ncic trial. Lancet Oncol (2009) 10(5):459–66. doi: 10.1016/s1470-2045(09)70025-7 19269895

[26] Stupp R Gander M Leyvraz S Newlands E. Current and future developments in the use of temozolomide for the treatment of brain tumours. Lancet Oncol (2001) 2(9):552–60. doi: 10.1016/s1470-2045(01)00489-2 11905710

[27] Denny BJ Wheelhouse RT Stevens MF Tsang LL Slack JA. Nmr and molecular modeling investigation of the mechanism of activation of the antitumor drug temozolomide and its interaction with DNA. Biochemistry (1994) 33(31):9045–51. doi: 10.1021/bi00197a003 8049205

[28] Ge X Pan M-H Wang L Li W Jiang C He J. Hypoxia-mediated mitochondria apoptosis inhibition induces temozolomide treatment resistance through mir-26a/Bad/Bax axis. Cell Death Dis (2018) 9(11):1128. doi: 10.1038/s41419-018-1176-7 30425242 PMC6233226

[29] Festuccia C Mancini A Colapietro A Gravina GL Vitale F Marampon F. The first-in-Class alkylating deacetylase inhibitor molecule tinostamustine shows antitumor effects and is synergistic with radiotherapy in preclinical models of glioblastoma. J Hematol Oncol (2018) 11(1):32. doi: 10.1186/s13045-018-0576-6 29486795 PMC5830080

[30] Hegi ME Liu L Herman JG Stupp R Wick W Weller M. Correlation of O6-methylguanine methyltransferase (Mgmt) promoter methylation with clinical outcomes in glioblastoma and clinical strategies to modulate mgmt activity. J Clin Oncol (2008) 26(25):4189–99. doi: 10.1200/jco.2007.11.5964 18757334

[31] Yu W Zhang L Wei Q Shao A. o6-methylguanine-DNA methyltransferase (mgmt): Challenges and new opportunities in glioma chemotherapy. Front Oncol (2020) 9:1547. doi: 10.3389/fonc.2019.01547 32010632 PMC6979006

[32] Yung WK Albright RE Olson J Fredericks R Fink K Prados MD. A phase ii study of temozolomide vs. procarbazine in patients with glioblastoma multiforme at first relapse. Br J Cancer (2000) 83(5):588–93. doi: 10.1054/bjoc.2000.1316 PMC2363506 10944597

[33] Stupp R Mason WP van den Bent MJ Weller M Fisher B Taphoorn MJ. Radiotherapy plus concomitant and adjuvant temozolomide for glioblastoma. New Engl J Med (2005) 352(10):987–96. doi: 10.1056/NEJMoa043330 15758009

[34] Henrichs B Walsh RP. Intraoperative mri for neurosurgical and general surgical interventions. Curr Opin anaesthesiol (2014) 27(4):448–52. doi: 10.1097/aco.0000000000000095 24848271

[35] Ginat DT Swearingen B Curry W Cahill D Madsen J Schaefer PW. 3 tesla intraoperative mri for brain tumor surgery. J magnetic resonance Imaging JMRI (2014) 39(6):1357–65. doi: 10.1002/jmri.24380 24921066

[36] Mislow JM Golby AJ Black PM. Origins of intraoperative mri. Magnetic resonance Imaging Clinics North America (2010) 18(1):1–10. doi: 10.1016/j.mric.2009.09.001 PMC4120097 19962089

[37] Kiesel B Freund J Reichert D Wadiura L Erkkilae MT Woehrer A. 5-ala in suspected low-grade gliomas: Current role, limitations, and new approaches. Front Oncol (2021) 11:699301. doi: 10.3389/fonc.2021.699301 34395266 PMC8362830

[38] Noh T Mustroph M Golby AJ. Intraoperative imaging for high-grade glioma surgery. Neurosurg Clin N Am (2021) 32(1):47–54. doi: 10.1016/j.nec.2020.09.003 33223025 PMC7813557

[39] Šteňo A Buvala J Babková V Kiss A Toma D Lysak A. Current limitations of intraoperative ultrasound in brain tumor surgery. Front Oncol (2021) 11:659048. doi: 10.3389/fonc.2021.659048 33828994 PMC8019922

[40] Olubiyi OI Lu F-K Calligaris D Jolesz FA Agar NY. Chapter 17 - advances in molecular imaging for surgery. In: Golby AJ, editor. Image-guided neurosurgery. Boston: Academic Press (2015). p. 407–39.

[41] Hadjipanayis CG Widhalm G Stummer W. What is the surgical benefit of utilizing 5-aminolevulinic acid for fluorescence-guided surgery of malignant gliomas? Neurosurgery (2015) 77(5):663–73. doi: 10.1227/NEU.0000000000000929 PMC4615466 26308630

[42] Barth CW Gibbs SL. Fluorescence image-guided surgery - a perspective on contrast agent development. Proc SPIE Int Soc Opt Eng (2020) 11222:112220J. doi: 10.1117/12.2545292 PMC7115043 32255887

[43] Stummer W Stocker S Wagner S Stepp H Fritsch C Goetz C. Intraoperative detection of malignant gliomas by 5-aminolevulinic acid-induced porphyrin fluorescence. Neurosurgery (1998) 42(3):518–25. doi: 10.1097/00006123-199803000-00017 9526986

[44] Gibbs SL. Near infrared fluorescence for image-guided surgery. Quant Imaging Med Surg (2012) 2(3):177–87. doi: 10.3978/j.issn.2223-4292.2012.09.04 PMC3496513 23256079

[45] Quek C Leong K. Near-infrared fluorescent nanoprobes for in vivo optical imaging. Nanomaterials (2012) 2(2):92–112. doi: 10.3390/nano2020092 28348298 PMC5327900

[46] Yamada T Signorelli S Cannistraro S Beattie CW Bizzarri AR. Chirality switching within an anionic cell-penetrating peptide inhibits translocation without affecting preferential entry. Mol Pharm (2015) 12(1):140–9. doi: 10.1021/mp500495u 25478723

[47] Lundberg P Langel Ü. A brief introduction to cell-penetrating peptides. J Mol Recognition (2003) 16(5):227–33. doi: 10.1002/jmr.630 14523933

[48] Trabulo S Cardoso AL Mano M De Lima MCP. Cell-penetrating peptides–mechanisms of cellular uptake and generation of delivery systems. Pharmaceuticals (2010) 3(4):961–93. doi: 10.3390/ph3040961 PMC4034016 27713284

[49] Kosuge M Takeuchi T Nakase I Jones AT Futaki S. Cellular internalization and distribution of arginine-rich peptides as a function of extracellular peptide concentration, serum, and plasma membrane associated proteoglycans. Bioconjugate Chem (2008) 19(3):656–64. doi: 10.1021/bc700289w 18269225

[50] Schneider AFL Kithil M Cardoso MC Lehmann M Hackenberger CPR. Cellular uptake of large biomolecules enabled by cell-surface-reactive cell-penetrating peptide additives. Nat Chem (2021) 13(6):530–9. doi: 10.1038/s41557-021-00661-x 33859390

[51] Derakhshankhah H Jafari S. Cell penetrating peptides: a concise review with emphasis on biomedical applications. Biomed Pharmacother (2018) 108:1090–6. doi: 10.1016/j.biopha.2018.09.097 30372809

[52] Teo SLY Rennick JJ Yuen D Al-Wassiti H Johnston APR Pouton CW. Unravelling cytosolic delivery of cell penetrating peptides with a quantitative endosomal escape assay. Nat Commun (2021) 12(1):3721. doi: 10.1038/s41467-021-23997-x 34140497 PMC8211857

[53] Erazo-Oliveras A Muthukrishnan N Baker R Wang T-Y Pellois J-P. Improving the endosomal escape of cell-penetrating peptides and their cargos: strategies and challenges. Pharmaceuticals (2012) 5(11):1177–209. doi: 10.3390/ph5111177 PMC3816665 24223492

[54] Yu S Yang H Li T Pan H Ren S Luo G. Efficient intracellular delivery of proteins by a multifunctional chimaeric peptide in vitro and in vivo. Nat Commun (2021) 12(1):5131. doi: 10.1038/s41467-021-25448-z 34446736 PMC8390694

[55] Laporte D Salin B Daignan-Fornier B Sagot I. Reversible cytoplasmic localization of the proteasome in quiescent yeast cells. J Cell Biol (2008) 181(5):737–45. doi: 10.1083/jcb.200711154 PMC2396804 18504300

[56] Dang FW Chen L Madura K. Catalytically active proteasomes function predominantly in the cytosol. J Biol Chem (2016) 291(36):18765–77. doi: 10.1074/jbc.M115.712406 PMC5009251 27417138

[57] Ma J Waxman DJ. Combination of antiangiogenesis with chemotherapy for more effective cancer treatment. Mol Cancer Ther (2008) 7(12):3670–84. doi: 10.1158/1535-7163.MCT-08-0715 PMC2637411 19074844

[58] Mehta RR Yamada T Taylor BN Christov K King ML Majumdar D. A cell penetrating peptide derived from azurin inhibits angiogenesis and tumor growth by inhibiting phosphorylation of vegfr-2, fak and akt. Angiogenesis (2011) 14(3):355–69. doi: 10.1007/s10456-011-9220-6 21667138

