# Supplementary figures and images for: Tumor-targeting cell-penetrating peptide, p28, for glioblastoma imaging and therapy
Source: Front Oncol. 2022 Jul 22;12:940001. doi: 10.3389/fonc.2022.940001 (PMC9353713; doi:10.3389/fonc.2022.940001)

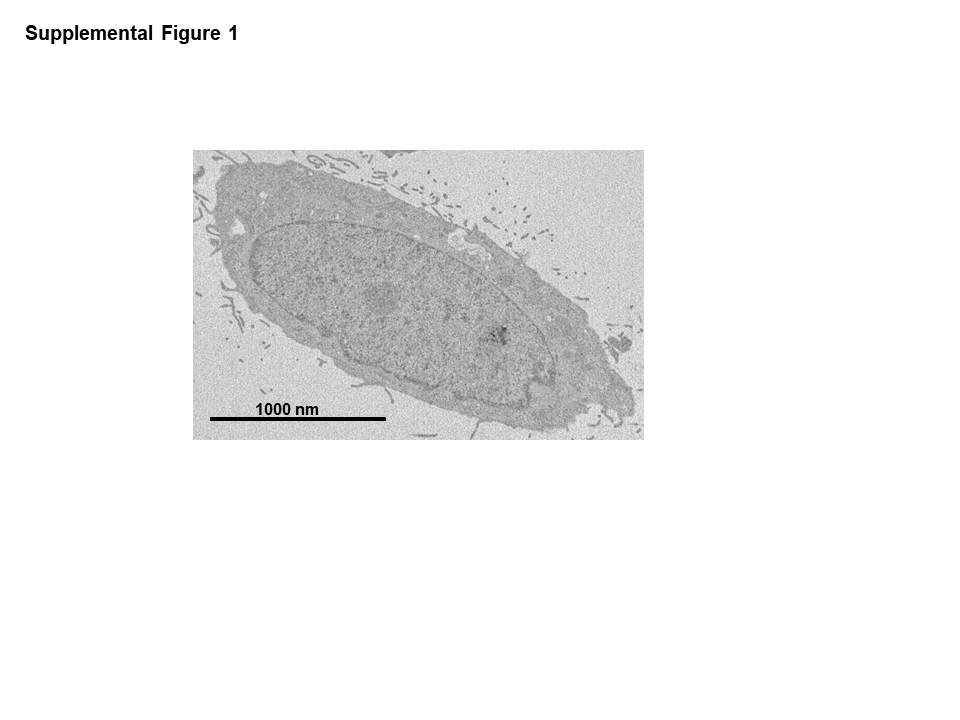

Supplement: Supplementary file 1 — Supplementary Figure 1 TEM image of GBM cells exposed to GNRs alone. LN-229 cells were treated with GNRs alone for 2 hr. Sections (60 nm) of fixed cells on 200 mesh copper grids were imaged. TEM images taken by a JEOL 1220 showed very little GNP cellular entry [file Image_1.tif]
